# The Investigation of the Binding of 6-Mercaptopurine to Site I on Human Serum Albumin

**DOI:** 10.1007/s10930-012-9449-y

**Published:** 2012-09-22

**Authors:** Jolanta Sochacka, Wojciech Baran

**Affiliations:** Department of General and Inorganic Chemistry, The School of Pharmacy and Division of Laboratory Medicine in Sosnowiec, Medical University of Silesia, ul. Jagiellońska 4, 41-200 Sosnowiec, Poland

**Keywords:** 6-Mercaptopurine, Human serum albumin, Site markers, Molecular docking

## Abstract

6-Mercaptopurine (6-MP) is one of a large series of purine analogues which has been found active against human leukemias. The equilibrium dialysis, circular dichroism (CD) and molecular docking were employed to study the binding of 6-MP to human serum albumin (HSA). The binding of 6-MP to HSA in the equilibrium dialysis experiment was detected by measuring the displacement of 6-MP by specific markers for site I on HSA, warfarin (RWF), phenylbutazone (PhB) and n-butyl *p*-aminobenzoate (ABE). It was shown, according to CD data, that binding of 6-MP to HSA leads to alteration of HSA secondary structure. Based on the findings from displacement experiment and molecular docking simulation it was found that 6-MP was located within binding cavity of subdomain IIA and the space occupied by site markers overlapped with that of 6-MP. Displacement of 6-MP by the RWF or PhB was not up the level expected for a competitive mechanism, therefore displacement of 6-MP was rather by non-cooperative than that the direct competition. Instead, in case of the interaction between ABE and 6-MP, when the little enhancement of the binding of ABE by 6-MP was found, the interaction could be via a positively cooperative mechanism.

## Introduction

6-Mercaptopurine (Purinethol, 6-MP) is one of synthetic analogues of natural purine bases, adenine and hypoxanthine, which is used in cancer chemotherapy [14]. This is indicated for remission induction and maintenance therapy of acute lymphocytic and lymphoblastic leukemia. 6-MP is also used as a treatment for inflammatory bowel such as Crohn`s disease. Clinical studies have shown that the absorption of an oral dose of 6-MP in humans is incomplete and variable, and associated with a half-life of 20–45 min in plasma, where it is bound to serum proteins averaging approximately 19 % of the administered dose [39]. The interaction of 6-MP with serum proteins was previously investigated [11, 45], but it has not been characterized in detail. Therefore it is of interest to describe interaction of 6-MP with human serum albumin (HSA), major protein component of blood plasma, to find the binding site for 6-MP in the protein tertiary structure.

HSA consists of tree homologous domains (I, II and III) and each domain is formed of two subdomains (A and B) [18]. The X-ray analysis of different ligand–HSA complexes showed the existence of two the most important binding sites in the II and III domains [3, 50]. Sudlow et al. [49] classified the two clearly separate binding sites for drugs: site I (warfarin binding site) and site II (benzodiazepine binding site). Fehske et al. [16] suggested two binding regions in site I: the warfarin and azapropazon binding regions. Moreover, Yamasaki et al. [56–58] proposed a concept that site I in HSA consists of three discrete binding regions, i.e. region Ia, Ib and Ic. Regions Ia and Ib correspond to the warfarin and azapropazon, respectively, and region Ic corresponds to n-butyl-*p*-aminobenzoate (ABE). The region Ia is partly overlapped with the region Ib and region Ic, and plays a bridge role between them. The alone Trp214 residue is located in the hydrophobic pocket in the binding subdomain IIA within the non-overlaping part of the warfarin region in which plays an important role in binding process of warfarin and acenocoumarol, whereas is not related to azapropazone binding region where phenylbutazone and other non-steroid anti-inflammatory drugs bind.

The investigations of drug–drug protein binding interactions showed a number of potential interactions between ligands that simultaneously bind to a protein. The binding of one drug can be inhibited by the presence of a second drug, and the mechanism may be either non-cooperative (competitive), when ligands bind to the same site or anti-cooperative when the binding of one ligand decreases the ability of the other ligand to bind. The others possibilities are: cooperative binding when the binding of one ligand facilitates the binding of the other and independent binding when the drugs bind independently and there is no interaction [2, 34]. Hence, the binding of one drug can be inhibited by the presence of a second drug not only by competition. Also the conformational changes in the albumin caused by second drug may decrease the binding of the first drug. The concept that much more subtle conformational change of protein accompanies binding of ligands was developed by a number of authors [23, 24, 30, 41, 42].

In this work the binding of 6-MP to site I of HSA was studied by equilibrium dialysis. The binding of 6-MP to this site was detected by measuring the displacement of 6-MP by specific site I marker ligands. In order to explain the observed experimental results and to have more detailed information about the specificity of the site for 6-MP the study was supplemented with computational method of molecular docking.

## Materials and Methods

### Chemicals

6-Mercaptopurine (6-MP), phenylbutazone (PhB), warfarin (RWF), n-butyl *p*-aminobenzoate (ABE) were purchased from Sigma Aldrich Chemical Co. Human serum albumin (HSA), fraction V, crystallized and lyophilized, was obtained from Biomed (Lublin, Poland). All other chemicals used were of analytical reagent grade.

### Solutions

The stock solution of 6-MP (1 × 10^−2^ M) was made by dissolving it in dimethyl sulfoxide. The solutions (1 × 10^−2^ M) of site markers RWF, PhB and ABE were prepared freshly in methanol. The final concentration of dimethyl sulfoxide and methanol in albumin solutions after addition of 6-MP and site markers solutions was smaller than 1 % v/v.

The albumin was dissolved in a 50 mM Tris–HCl buffer pH 7.4 and diluted to the required concentration. The concentration of the obtained solutions of albumin was determined spectrophotometrically using an molar extinction coefficient of 3.66 × 10^4^ L mol^**−**1^ cm^−1^ at 278 nm [38]. The molecular mass of HSA was assumed to be 66 500 Da.

### CD Measurements

CD measurements were carried out with a Jasco J-810 spectropolarimeter. Far UV-CD (190–250 nm) spectra were collected at 23 °C using a quartz cell with a path length of 0.02 cm at 0.2 nm intervals. The spectra were taken at protein concentration of 1 × 10^−5^ M in Tris–HCl buffer, pH 7.4. The residue ellipticity, [Θ]_MRW_ (deg cm^2^ dmol^−1^), was calculated from the measured Θ by using equation [43]:

$$ [\Uptheta ]_{\text{MRE}} = \frac{{\Uptheta \times 100 \times {\text{M}}_{\text{r}} }}{{{\text{c}} \times {\text{l}} \times {\text{N}}_{\text{A}} }} $$

where Θ is the measured ellipticity in degrees, c is the protein concentration in mg ml^−1^, l is the path length in cm, and M_r_ is the protein molecular weight, N_A_ is the number of amino acids (585 amino acids calculated from the primary structure) per protein.

The CD data were used to determine the relative amounts of the secondary structural elements of a protein using CDNN CD 2.1 software [12]. The procedure was based on the approximation that a protein CD spectrum in the amide region can be represented as a linear combination of the contribution of the different elements of secondary structure, according to equation [43]:

$$ \left[ {\Uptheta \left( \lambda \right)} \right] = {\text{f}}_{{{\upalpha}}} \times \left[ {\Uptheta_{{{\upalpha}}} (\lambda )} \right] + \left[{\text{f}}_{\text{t}} \times \left[ {\Uptheta_{\text{t}} (\lambda )} \right] + {\text{f}}_{\text{n}} \times \left[ {\Uptheta_{\text{n}} (\lambda )} \right]\right] $$

Where [Θ_α_(λ)], [Θ_t_(λ)] and [Θ_n_(λ)] are the basis spectra for α-helical, turn and non-regular structures.

### Equilibrium Dialysis

Equilibrium dialysis experiment was performed with bags of 5 cm length made from cellulose tubing, 25 mm diameter when inflated (Dialysis Tubing Cellulose Membranes with molecular weight cut-off of 12 kDa, Sigma Aldrich Co). The bags were previously prepared as suggested by the manufacturer and then they were conditioned in Tris–HCl buffer pH 7.4 for 30 min before used. 4 mL of albumin was pipetted into a bag (compartment A) which was placed in a 10 mL glass tube containing 4 mL free-protein buffer and separately RWF, PhB, ABE or 6-MP, and mixture of 6-MP–RWF, 6-MP–PhB or 6-MP–ABE (compartment B). The tubes were closed with parafilm, and were stirred constantly at 20 rotations per minute, at 23 °C. A solution of drug(s) in the compartment B and a solution of albumin in the compartment A were allowed to equilibrate. In the preliminary experiments it was found that equilibrium was established after 12 h of dialysis in case of all ligands, so all samples were measured after this equilibrium time. Control experiments in the absence of albumin showed that the effect of ligands adsorption on tubes and onto membrane was negligible and analytical recovery of ligands in the system was about 98 %. The volumes of the solutions in two compartments remained constant during the dialysis procedure and were not corrected. Experimental measurements with standard deviation higher than 10 % were rejected. Dialysis with each ligand or mixture of ligands was performed in fivefold and the average value of *C*_b_ was used as binding data. Protein concentration was 1 × 10^−5^ M and ligands concentration was 0.8 × 10^−5^ M.

The free-ligands concentrations in compartment B at the beginning of the dialysis and after equilibrium were determined by HPLC. The HPLC system consisted of the Knauer HPLC pump 64 controlled by the LP-Chrom v.1.0 computer software and the UV Waters TAD 486 variable wavelength detector. A column of LiChrospher 100 RP-18 (5 μm in 4 × 250 mm) (Merck KGaA) was used as the stationary phase. The mobile phase consisted of methanol–sodium acetate buffer pH 4.5 (77:23, v/v) for 6-MP, acetonitryl–phosphate buffer pH 7.7 (20:80, v/v) in the case of RWF and PhB and acetonitryl–deionized water (70:30, v/v) for ABE. The mobile phase was run at the rate of 1 ml/min. 6-MP was detected at 330 nm and RWF, PhB and ABE were detected at 310, 280 and 270 nm, respectively. All solvents were filtered and degassed prior to use. The injection volume was 50 μL in all the cases. The free ligand concentration was determined using a calibration curve obtained from the concentration of dialyzed ligand in the absence of albumin.

The bound ligand concentration (C_b_) was calculated by estimation of free-ligand concentration (C_f_) according to the equation [57]:

1 $$ {\text{C}}_{\text{L(b) }} = {\text{C}}_{\text{L(0)}} - 2 \times {\text{C}}_{\text{L(f)}} $$

where C_L(b)_ was concentration of bound ligand, C_L(f)_ was final concentration of ligand in buffer compartment and C_L(0)_ was starting concentration of ligand (total ligand concentration before equilibrium dialysis).

The free fraction f_(f)_ was given by [21]:

2 $$ {\text{f}}_{{ ( {\text{f)}}}} = {\text{C}}_{\text{L(f)}} / ( {\text{C}}_{\text{L(0)}} - {\text{C}}_{\text{L(f)}} ) $$

and the bound fraction f_(b)_ was:

3 $$ {\text{f}}_{{({\text{b}})}} (\% ) = (1 - {\text{f}}_{{ ( {\text{f)}}}} ) \times 100 $$

Displacement percentage (p%) [56] of 6-MP and increase in free fraction (Δ%) of 6-MP by site markers were given by:

4 $$ {\text{p(}}\% )= [({\text{f}}_{\text{b(1)}} - {\text{f}}_{{{\text{b}}(2)}} )/{\text{f}}_{{{\text{b}}(1)}} ] \times 100 $$

5 $$ \Updelta (\% ) = [({\text{f}}_{\text{f(2)}} - {\text{f}}_{\text{f(1)}} )/{\text{f}}_{\text{f(1)}} ] \times 100 $$

where f_b(1)_ and f_b(2)_ were the bound fractions of 6-MP without and with markers (displacer), respectively; and f_f(1)_ and f_f(2)_ were the free fractions of 6-MP without and with markers (displacer), respectively.

The HSA binding data (f_b_) were converted into an equivalent binding affinity log K_a_^HSA^ with the following equation derived from the law of mass. The log K_a_^HSA^ was the binding affinity to HSA under the assumption that binding occurs exclusively to HSA, a binary complex was formed, and an excess of protein was present compared to the concentration of the ligands [32]:

6 $$ {\text{logK}}_{\text{a}}^{\text{HSA}} = [{ \log }\left( {{\text{f}}_{\text{b}} /1 - {\text{f}}_{\text{b}} } \right)] - {\text{logC}}_{\text{HSA}} $$

where C_HSA_ was the concentration of HSA (1 × 10^−5^ M).

The percent binding before and after displacement was compared by using a two-way analysis of variance for balanced data. The confidence intervals based on the residual variance were computed for each pairs of bound fraction before and after displacement.

### Molecular Docking

Molecular docking procedure was performed using the Molegro Virtual Docker (MVD) program [37, 52]. X-ray structure of HSA (PDB ID: 1AO6) [7] was downloaded from Protein Data Bank (PDB). The ionizable residues were set to their pH 7.4 protonation states; the His, Arg and Lys were protonated, while those of Asp and Glu were deprotonated. The crystallographic water molecules were removed from the protein. The identification of the cavity with the potential binding site for ligands in subdomain IIA in HSA crystal structure (1AO6.pdb) was performed automatically using the grid-based cavity prediction algorithm. The residues close to cavity were minimized. During the minimization only torsion angles in the sidechains were modified, all other properties (including bond lengths and backbone atom positions) were hold fixed.

**Fig. 1:**
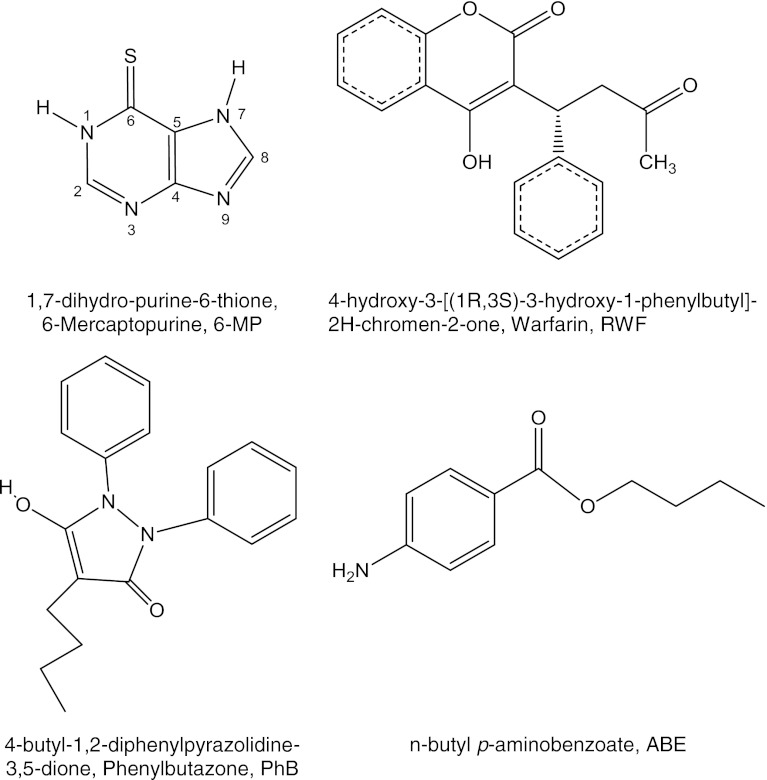
Chemical structures of 6-MP and site markers

The two dimensional (2D) structures of 6-MP and ABE were obtained using the ChemDraw Ultra [4] program. 2D structures were converted to three dimensional (3D) representations by the use of CS Chem3D Ultra [8] program and the geometry of the molecules was energy-minimized using semiempirical (AM1) method implemented in the same software, and molecules were imported to MVD as.mol files. The initial conformation (3D structure) of warfarin was extracted from RWF–HSA complex (PDB ID: 2BXD) [47] and phenylbutazone from PhB–HSA complex (PDB ID: 2BXP) [19] and each was once more energy-minimized. The chemical 2D and 3D structures of all ligands are shown in Fig. 1.

During the docking simulation the backbone was kept rigid, but the torsional angles in the side chains of amino acids close to the detected cavity were allowed to change. The following steps were applied during the docking simulation: the ligands were docked with the softened potentials. At this point the receptor was kept rigid at its default conformation. After each ligand was docked, the sidechains chosen for minimization were minimized with respect to the found pose. After repositioning the sidechains, the ligand was energy-minimized. The repositioning of the sidechains and minimization of the ligand were performed using the standard non-softened potentials. All flexible torsions in the ligand were set rigid during docking, because the complexity of the docking search can be significantly reduced, if the number of torsions that are set flexible during the docking run is lowered.

First, to obtain the 6-MP–HSA and markers–HSA complexes, the 6-MP molecule as neutral and monoanionic forms, RWF and PhB as monoanionic forms, and neutral molecule of ABE were docked individually to the cavity. For each complex 10 independent runs were conducted, each of these runs was returning to a single final solution (pose). The resulting conformations were clustered and only the negative lowest-energy representation from each cluster was returned when the docking run was completed; the similar poses were removed keeping the best-scoring one. The cluster of ten poses was sorted in order of the Rerank Score. For analysis one pose with lowest value of Rerank Score was selected as the best solution for each complex. Next, the same procedure was followed in order to obtain the RWF–6-MP–HSA, PhB–6-MP–HSA and ABE–6-MP–HSA complexes. The both forms of 6-MP were docked one at a time into cavity when RWF, PhB or ABE were earlier bound.

Before docking of the ligands into the HSA structure, the MVD docking protocols were validated using the two crystal structures of RWF/HSA and PhB/HSA complexes. Both the X-ray complexes were taken from PDB: RWF/PDB ID: 2BXD [47] and PhB/PDB ID: 2BXP [19]. In both complexes the ligands were in the unionized forms. For each individual in the initial population the cocrystallized reference ligand was selected as center of search space and one cavity, restricted to search space, was detected. Each ligand was extracted from its original PDB X-ray structure and the remaining HSA structure was used as a docking simulation template. Protein was previously prepared and the incomplete amino acid residues with structural errors were reconstructed and optimized. The re-docking procedure was conducted; RWF was re-docked to the RWF binding site 2BXD and PhB to the binding site 2BXP. RMSD (the root mean square deviation) threshold for multiple cluster poses was set at <2 Å. The docking simulations results were compared with the complexes structures determined in X-ray crystallography experiments. In the crystal structures, RWF and PhB bind in one site in subdomain IIA. RWF has hydrogen bonds with Tyr150 and His242 (interactions for instance 2BXD:RWF:A:2001 generated by Pose View Software, RCSP PDB) and PhB making H-bonds with Arg218 and His242 (interactions for instance 2BXP:P1Z:A:3001 generated by Pose View Software, RCSP PDB). By comparison the interactions between ligands (RWF or PhB) and HSA in the complexes from MVD with those for the crystal structures of ligand/HSA complexes, it was found that the RWF and PhB have some interactions in common with those of these ligand and that binding modes were reproduced in re-docking test. The obtained RMSD (the root mean square deviation from a reference ligand, in Å) values between crystal and docked poses of the RWF and PhB were 2.00 and 1.08 Å, respectively. Hence, it was proved that MVD may be used as a reliable tool for predicting the binding site and binding mode of 6-MP in this study.

### Physicochemical Properties of 6-MP and Site Markers

The thione–thiol tautomerism and protropic tautomerism in imidazole moiety can occur simultaneously in 6-MP and thus two types of equilibrium can be observed: thiol ↔ thione and N(7) ↔ N(9). However, thione-N1, 7(H) tautomer is preferred in an aqueous solution [6]. 6-MP is acidic compound. The N(1)H group in pyrimidine ring is more acidic than the NH group in the imidazole ring, therefore the first proton is removed from the N(1) group [33]. The physicochemical properties (p*K* and log K_a_^HSA^) of 6-MP and marker ligands were estimated using ACD/Labs software (Advanced Chemistry Development, Inc.) [20]. The dissociation constants and the fractions of the ionic species having a particular net charge at pH 7.4 were estimated using prediction module p*K*_a_. In aqueous solution at pH 7.4, 6-MP occurs as a mixture of the neutral and monoanionic form (40.3 % ionized form) owing to the fact that its p*K* value is 7.72. The RWF and PhB are also acidic compounds. Their p*K* value is 4.3 and they will be fully ionized in aqueous solution at pH 7.4. The ABE is a weak base and in solution at pH 7.4 exists as 100 % non-charged molecule. It was calculated by physicochemical properties module, that 6-MP and ABE are good hydrogen–bond donors and acceptors and RWF and PhB must be only hydrogen–bond acceptors in ionized state. The equilibrium binding constants to HSA (log K_a_^HSA^) were estimated using prediction module distribution/protein binding in this software. The calculated values of binding constant were 5.19, 5.39 and 3.62 for PhB, RWF and ABE, respectively and 3.09 for 6-MP.

## Results

### CD Spectroscopy Studies

The CD spectrum of protein in the far-UV spectral region (190–250 nm) originates from the dichroic absorbance of the amide bonds, and therefore this region is termed the amide region. The CD in amide region reports on the backbone (i.e. secondary) structure of a protein, in particular α-helix, and is used to characterize the secondary structure of protein and change therein [43]. The crystallographic analysis of HSA shows that the protein is 67 % α-helical and contains no β-sheet. The remaining 33 % of albumin consists of polypeptides occurring in turns and extended or flexible regions between subdomains. Although β-sheet is absent in the structure of serum albumin, about 23 % of the structure is in an extended chain conformation, which would be predicted as β-strand, and about 10 % of the remaining structure exists in turns [3, 9, 10, 18]. The content of α-helix depends on structural arrangements of the HSA in the solid state and aqueous solutions, therefore the results from X-ray structural analysis and CD measurements may differ. The interaction between ligand and protein usually induces perturbations in the structure of protein mainly at the binding site. Structural changes in protein caused by the binding of ligands were reported by number of authors [25, 26, 36, 53]. The alteration in protein conformational stability upon protein–ligand interactions, could be determine by helical content using the CD, because the α-helix displays a strong and characteristic CD spectrum in the far-UV region.

**Fig. 2:**
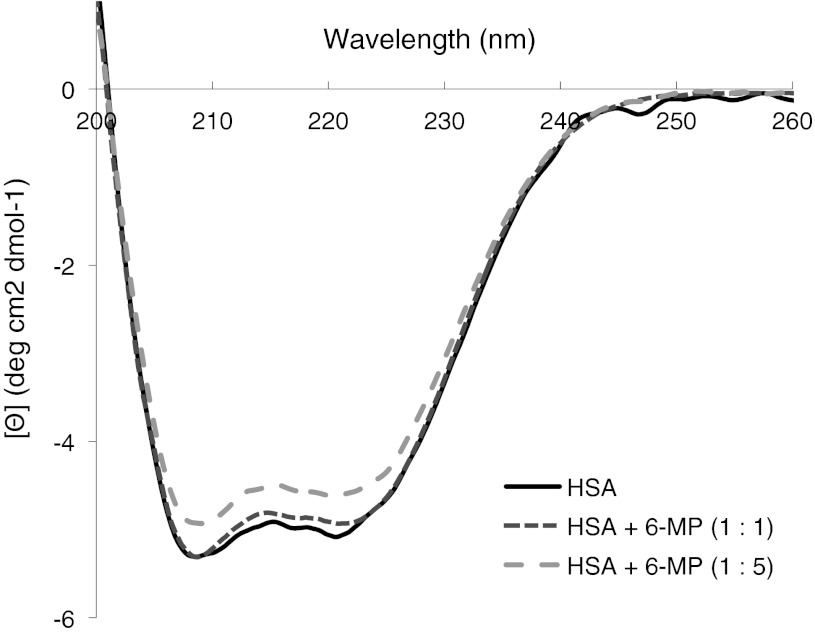
CD far-UV spectra of HSA in the native state and in the presence of 6-MP. The experiment was carried out in Tris–HCL buffer, pH 7.4. Concentration of HSA was 1 × 10^−5^ M, and concentrations of 6-MP were 1 × 10^−5^ M and 5 × 10^−5^ M (i.e. molar ratios HSA: 6-MP, 1:1 and 1:5, respectively)

The far UV-CD spectroscopy was used for determination of effect of 6-MP on the secondary structure of HSA. As Fig. 2 shows the CD spectrum of native HSA exhibits two negative bands in the far UV region at 208 nm and 222 nm characteristic of an α-helical structure of protein. The helical content calculated by CDNN CD 2.1 software [12] for HSA alone at pH 7.4 was found to be 55.0 % (Table 1), which was in agreement with earlier reports [48, 51]. Upon addition of 6-MP to the solution of HSA (molar ratios HSA : 6-MP, 1:1 and 1:5), the intensity of both of these bands changed distinctly as compared to the native structure of HSA at pH 7.4. Secondary structural elements calculated by using the CDNN program showed that the α-helix structure decreased by approximately 4 % for equimolar solutions and 9 % for solutions with higher concentrations of 6-MP (Table 1). From the above results it is apparent that the secondary structure of HSA after addition of 6-MP was also predominantly α-helix, but it becomes partially disordered with the loss of helical content. The observed change could be the result of the formation of complex between HSA and 6-MP.

**Table 1:** Secondary structure components of HSA in native state and in presence of 6-MP

Secondary structural elements of a HSA	HSA	HSA + 6-MP (1:1)	HSA + 6-MP (1:5)
α-Helix (%)	55.0 ± 1.0	53.0 ± 0.5	50.0 ± 1.1
Antiparalel (%)	4.4 ± 0.4	4.5 ± 0.4	4.9 ± 0.5
Parallel (%)	4.7 ± 0.1	5.3 ± 0.2	5.7 ± 0.2
β-turn (%)	13.6 ± 0.8	14.0 ± 1.0	14.2 ± 0.9
Random coil (%)	20.8 ± 1.0	21.6 ± 1.3	23.6 ± 1.2

Each value is the mean ± SD of data from three determination. Based on the Fig. 2, the data were analyzed by CDNN 2.1 software [12]

### Effect of Site Markers on 6-MP Binding to HSA

Equilibrium dialysis was carried out in order to identify the binding site of 6-MP in HSA structure. In the experiments the RWF, PhB and ABE were employed as the site markers that bind specifically to site I of HSA; RWF and PhB were used as the markers for warfarin–region and for azapropazone–region binding of drug, respectively and ABE as marker which represents binding region Ic located adjacent to the warfarin binding region but apart from that of azapropazone [57]. Individual binding of the 6-MP and markers to HSA, and simultaneous binding of three pairs 6-MP–RWF, 6-MP–PhB and 6-MP–ABE to HSA was studied. Due to the differences in the displacement of 6-MP by the markers it was determined to which region(s) of site I 6-MP may bind. The percentage of binding of each ligand with and without the presence of other ligand, and the percentage of 6-MP displacement are shown in Table 2. From these results the primary association constant log K_exp_^HSA^ for each ligand before displacement was also determined. Because for most of the drugs with high binding affinity to serum protein, the primary association constant is well separated from the following binding constants, that the first association constant is the most relevant one from a pharmacological point of view [32]. The (log K_exp_^HSA^) values obtained by equilibrium dialysis were used to compare the data for the 6-MP and site markers. In addition, it was confirmed that also at ligand HSA ratio <1 the ligand binds to HSA with high ability. The log K_exp_^HSA^ values for RWF, PhB and ABE were higher from the literature values (log K_lit_^HSA^) [32]. The differences from published values were probably due to the distinct conditions which influence ligand binding, for instance albumin lot, pH temperature and technique. The log K_calc_^HSA^ value for the RWF, PhB and 6-MP which was calculated by I-Lab software [20] was significantly lower than that obtained by equilibrium dialysis. This difference may stem from the fact that the software calculates the log K_calc_^HSA^ only for the neutral molecule.

The results of the displacement experiment are summarized in Table 2. At equilibrium concentrations of ligands in the albumin during individual binding, the 6-MP was bound to HSA

**Table 2:** Bound fraction (f_b_%) and affinity constant (log K^HSA^) of 6-MP and site markers to HSA determined by equilibrium dialysis

System	Before displacement				Primary association constant (log K^HSA^)		
	Bound ligand fraction (f_b_%)						
	RWF	PhB	ABE	6-MP	log K_exp_^HSA^	log K_lit_^HSA^	log K_calc_^HSA^
RWF-HSA	84.7 ± 0.7				5.74	5.33	5.39
PhB-HSA		83.4 ± 1.3			5.70	5.54	5.44
ABE-HSA			37.3 ± 0.7		4.77	4.45	3.62
6-MP-HSA				23.3 ± 1.5	4.48	*	3.09

System	After displacement				Displacement of bound 6-MP p(%)	Increase in free fraction of 6-MP Δ(%)
	Bound ligand fraction (f_b_%)					
	RWF	PhB	ABE	6-MP		
6-MP-RWF-HSA	86.3 ± 0.8			18.1 ± 0.7	22.3 ± 0.8	6.8 ± 0.7
6-MP-PhB-HSA		83.6 ± 1.9		19.0 ± 0.9	18.5 ± 0.9	5.6 ± 1.0
6-MP-ABE-HSA			39.5 ± 0.6	23.7 ± 0.8	<1.0	<1.0

(f_b_%) calculated from the Eq. (3), (p%) calculated from the Eq. (4), calculated from the Eq. (5), experimental log K_exp_^HSA^ calculated from the Eq. (6), log K_lit_^HSA^ data from the literature [32], log K_calc_^HSA^ data from the I-Lab software [20], * no literature data could be found. Concentration of HSA was 1 × 10^−5^ M and concentrations of ligands before the equilibration were 0.8 × 10^−5^ M. Each value is the mean ± SD of data from three determination

to a lesser degree than the other three, only by ~23.3 %. When the RWF and PhB were added to the 6-MP–HSA complex, the bound 6-MP fractions were even lower as they decreased to ~18.0 %, while 6-MP binding was not affected by ABE binding. This showed that the markers caused different effects on 6-MP binding to HSA. The binding of 6-MP was inhibited by the RWF or PhB, but no mutual displacements were observed, because the bound RWF and PhB fractions were practically unchanged by 6-MP. The bound fraction (f_b_%) of 6-MP was assumed to be the same in the absence and presence of ABE, whereas the binding of ABE was enhanced by the addition of 6-MP, resulting in a 3.5 % (p < 0.05) decrease of free fraction (Δ%) in the proportion of ABE alone.

### Prediction of 6-MP Binding to Site I and Comparison of 6-MP Binding with the Site Markers in the Complex

The molecular docking simulation using MVD program [37] was employed to examine the binding mode of 6-MP with HSA. Based on the experimental data from equilibrium dialysis and displacement experiment it was concluded, that 6-MP may bind to the site I in the HSA molecule similarly as RWF, PhB and ABE. Therefore, the both 6-MP and markers were docked to cavity determined in site I. First, individual docking of the ligands was studied. Total binding energy between selected conformation of each ligand and HSA, and components of this energy determined from MVD results are listed in Table 3. The results from Table 3 were interpreted on the assumption that the more negative are the values of predicted binding energy, the more thermodynamically favorable is binding energy [1]. When the 6-MP and markers were docked individually into cavity, all the 6-MP–HSA complexes, taking into account E-Inter values, exhibited less favorable interactions with HSA than these of RWF, PhB and ABE. It was also observed, that binding energy between 6-MP and HSA depends on the 6-MP ionization state, and was stronger for anionic form that unionized 6-MP molecule. The estimated E-Total binding energy for all the ligands was strongly dependent on steric interactions. Hydrogen bonds and electrostatic interaction energies accounted for markedly smaller proportion of total binding energy (Table 3, part A).

**Table 3:** Binding energies of 6-MP and site markers at the binding site I on HSA

Ligand (pose)	MolDock score energy (arbitrary units)							
	Rerank score	E–total	E–inter	Steric	Hbonds	Electro	Electro–long	E–intra
A Ligands individually docked to cavity								
PhB (anion)	−100.5	−128.4	−130.6	−114.5	−5.0	−7.4	−3.7	2.2
RWF (anion)	−30.3	−108.2	−127.3	−118.4	−6.1	−1.4	−1.4	19.1
ABE (neutral)	−75.2	−88.8	−94.2	−91.1	−3.1	0.0	0.0	5.4
6-MP (neutral)	−56.5	−66.1	−69.7	−63.5	−6.2	0.0	0.0	3.7
6-MP (anion)	−62.3	−72.8	−76.5	−57.0	−6.7	−11.6	−1.2	3.7
B 6-MP docked to RWF–HSA complex								
6-MP (neutral)	−53.0	−66.8	−70.5	−68.6	−1.9	0.0	0.0	3.7
6-MP (anion)	−56.7	−71.6	−75.3	−60.9	−7.1	0.0	−7.3	3.7
C 6-MP docked to PhB–HSA complex								
6-MP (neutral)	−48.3	−63.3	−66.9	−58.7	−8.2	0.0	0.0	3.7
6-MP (anion)	−46.6	−68.0	−71.7	−61.0	−3.2	−4.2	−3.3	3.7
D 6-MP docked to ABE–HSA complex								
6-MP (neutral)	−55.5	−64.3	−68.0	−60.7	−7.3	0.0	0.0	3.7
6-MP (anion)	−57.5	−71.8	−75.5	−59.3	−7.2	−7.5	−1.5	3.7
E ABE docked to 6-MP–HSA complex								
ABE (neutral)	−74.6	−89.1	−94.6	−86.5	−8.1	0.0	0.0	5.5

The score is not normalized in chemical units and should only be used to compare the results within one results set. E-Total—the total MolDock Score energy (the sum of internal ligand energies, protein interaction energies and soft penalties), E-Inter—the MolDock Score interaction energy between the pose and the protein (equal to Steric + HBond + Electro + ElectroLong), HBond—hydrogen bonding energy between protein and ligand, Steric—steric interaction energy between protein and ligand, Electro—the short-range (r < 4.5 Å) electrostatic protein–ligand interaction energy, ElectroLong—the long-range (r > 4.5 Å) electrostatic protein–ligand interaction energy, E-Intra—the total internal MolDock Score energy of the pose

The inside wall of the binding pocket of subdomain IIA is formed by group of hydrophobic side chains of amino acids (Phe211, Trp214, Ala215, Leu219 etc.). The apolar interior contains two clusters of polar residues, one towards the bottom of the pocket (Tyr150, His242, Arg257) and second surrounding the entrance of the pocket (Lys195, Lys199, Arg218 and Arg222). The Arg218 and Arg222 are partially solvent exposed, while Arg257 is buried in the hydrophobic core of HSA [17, 50]. The residue of Lys199 is situated in the vicinity of the hydrophobic pocket and its side chain pointing towards the entrance channel of the binding crevice. The residue of Lys195 is placed at the opening of the IIA cavity and forms a salt bridge with Glu292, which is close to other charged residues as Arg218, Arg222, etc. [13].

**Fig. 3:**
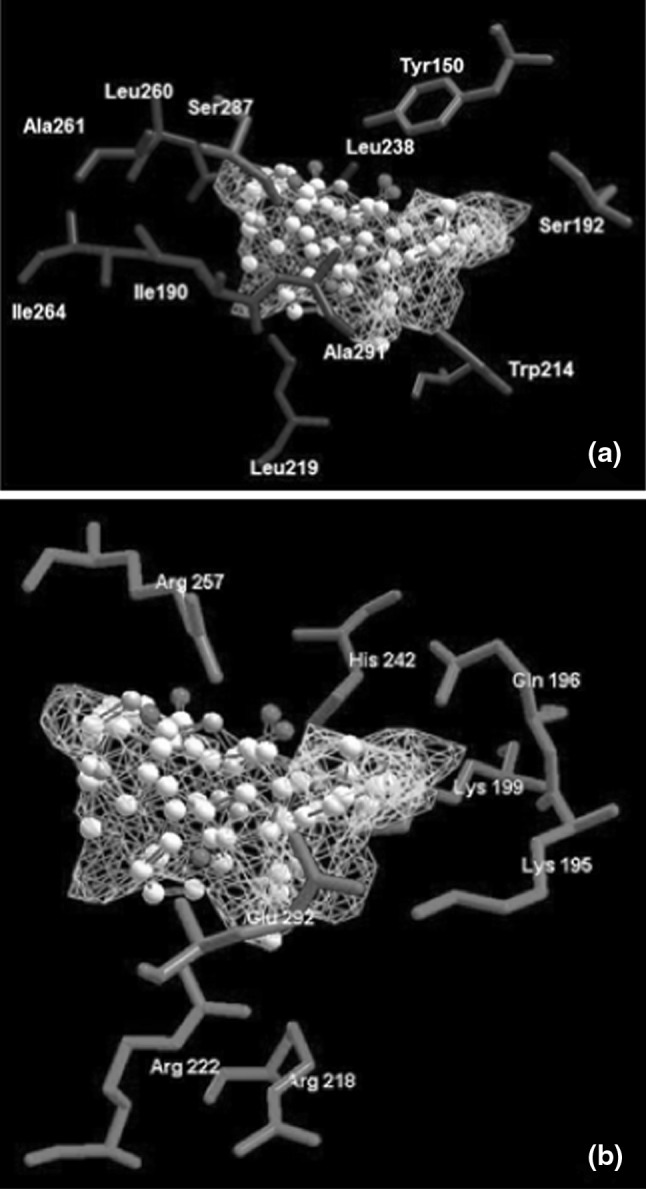
The close-up of the detected cavity with a hydrophobic (**a**) and hydrophilic (**b**) amino acids residues capable of interacting with 6-MP and site markers (RWF, PhB and ABE), in stereo. Only residues around 6 Å of the docked ligands are displayed. Amino acids residues are *colored* according to the hydropathy index [27], *violet* is interpolated between red for the residues with high hydrophobicity and blue for the residues with high hydrophilicity, the cavity is depicted in *green mesh*. The ligands docked into cavity are shown in a *ball* and *stick* representation and amino acids in a *stick* representation. The all hydrogen were hidden for clarity (Color figure online)

**Fig. 4:**
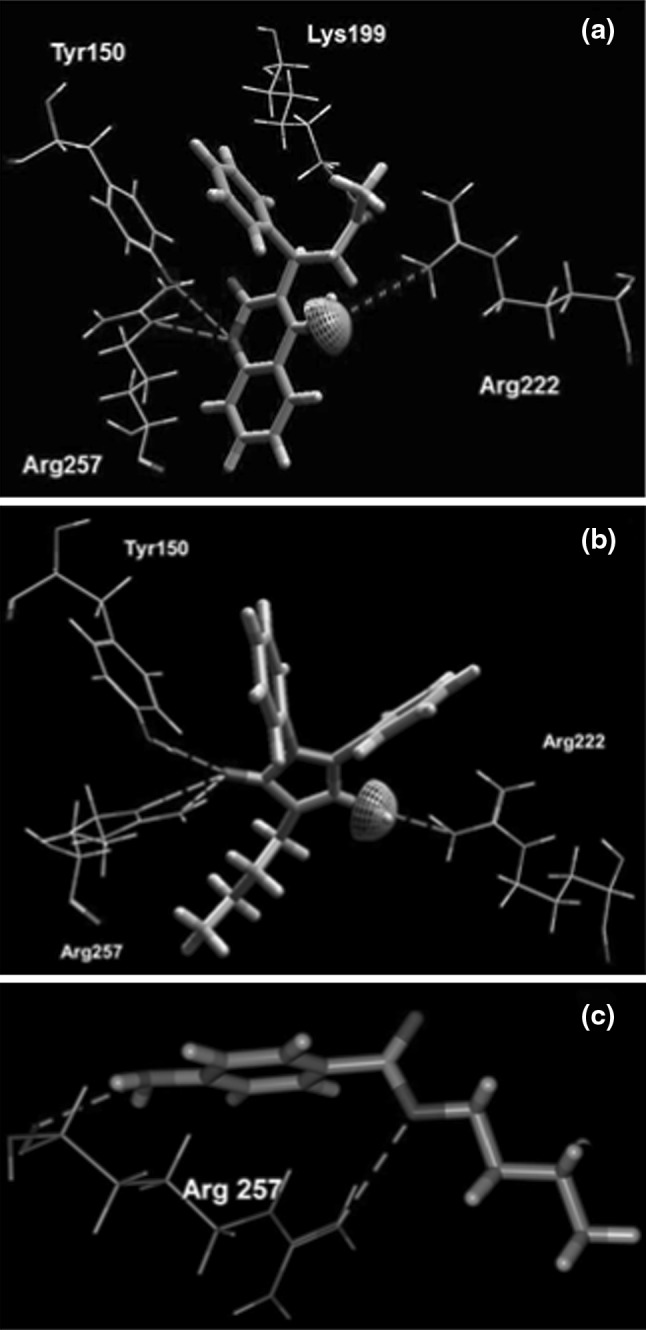
Superposition of the binding mode of RWF (**a**), PhB (**b**) and ABE (**c**) from the HSA–marker complexes structures obtained by molecular docking. Hydrogen bonding interactions are shown in *dashed green lines*, strong electrostatic interactions are visualized as partial spheres oriented in the direction of the interaction and as *dashed green lines*. The ligands and amino acids are shown in a *stick* and *thin stick* representation, respectively. The ligands and amino acids are shown in a *stick* and *thin stick* representation, respectively and are *colored* according to element type (in the CPK* color* scheme) (Color figure online)

Figure 3 presents the close-up of the detected cavity with a non-polar (Fig. 3a), and polar and charged (Fig. 3b) amino acids residues capable of interacting with ligands. When analyzing the best energy ranked interactions between markers and amino acids it can be seen that the residues of Tyr150, Lys199, Arg222 and Arg257 in the vicinity of RWF (Fig. 4a) and PhB (Fig. 4b) were in suitable position to make hydrogen bond interactions with the both markers. The positively charged guanidinium group of Arg222 residue and ε-amino NH_3_^+^ group of Lys199 were able to form electrostatic (ionic) interactions with negatively charged oxygen atom of the ligands moiety of RWF and PhB, respectively. These and other hydrophobic (Leu238, Leu260, Ile264, Ile290, Ala291, Ser292, Ser287) and hydrophilic (Lys195, His242, Glu292) residues which were the part of the binding site (Fig. 3a, b) could make steric interactions with ligands. Results obtained from the bonding interaction of ABE to HSA indicated that ABE was bound only via two weak hydrogen bonds between oxygen (as an acceptor) in ABE and guanidinium group of Arg257, and between secondary amine group (as an donor) in ABE and main chain carbonyl oxygen of Arg257 (Fig. 4c). The steric interaction with Trp214 residue was observed only in case of RWF.

**Fig. 5:**
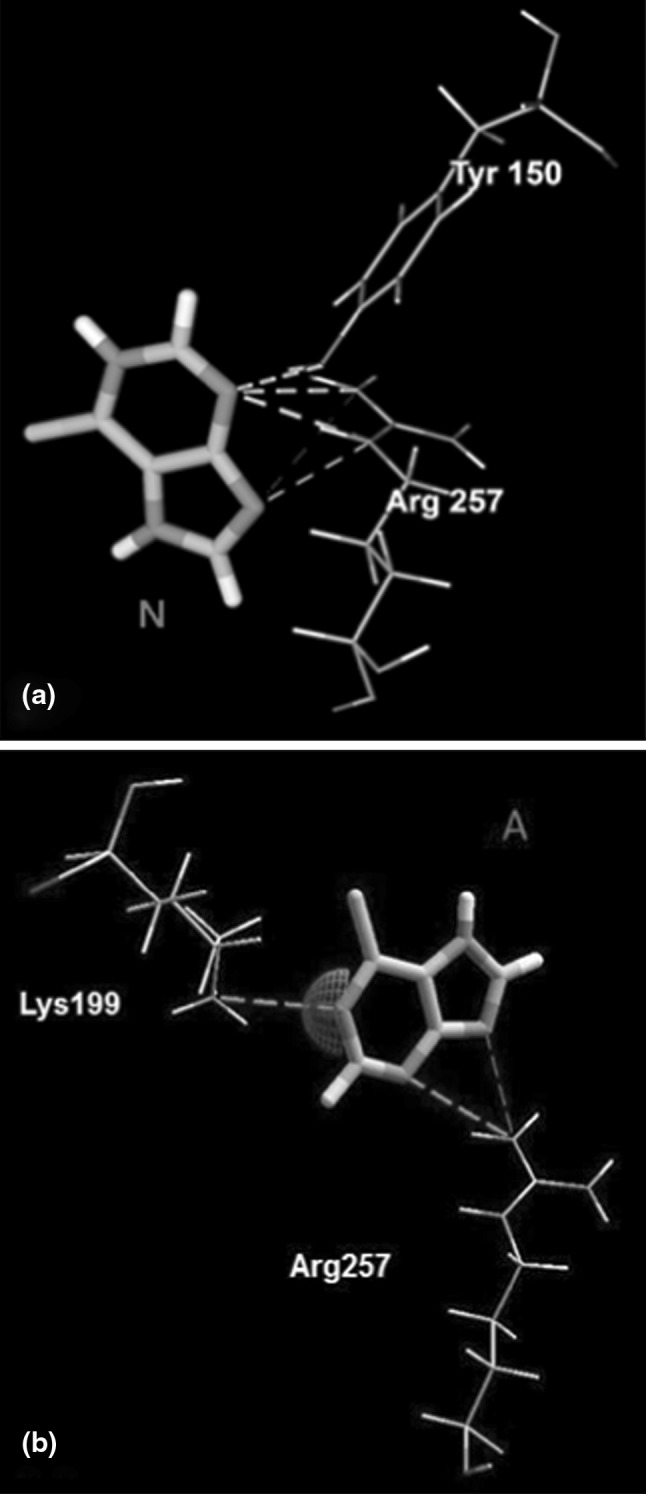
Superposition of the binding mode of neutral form of 6-MP (**a**) and anionic (**b**) forms of 6-MP from the HSA–6-MP complexes structures obtained by molecular docking using MVD program. Hydrogen bonding interactions are shown in *dashed green lines*, strong electrostatic interaction is visualized as partial sphere oriented in the direction of the interaction and as *dashed green line*. The ligands and amino acids are shown in a *stick* and *thin stick* representation, respectively and are *colored* according to element type (in the CPK *color* scheme). N and A, neutral and anionic form of 6-MP, respectively (Color figure online)

Detailed warfarin and phenylbutazone binding studies by the use fluorescence, absorption spectrometry and near-UV circular dichroism showed that the both of the ligands bind with high-affinity to a region in subdomain IIA [5, 22, 35, 54, 55]. The X-ray crystalographic analysis of warfarin–HSA and phenylbutazone–HSA complexes provided the more information about the interactions between those drugs and HSA in binding site. It was found that warfarin and phenylbutazone interact with Lys195, Arg219, Arg222, His242 and Arg257 and that in native protein at pH 7.4, when warfarin is negatively charged, the ionic interaction with Arg218 is most probable. Watanabe et al. [54] found that the Trp214 could be part of the high-affinity binding site of warfarin, but is does not seem to be essential for binding. Comparing the interaction of the RWF and PhB with HSA in the complexes obtained by molecular docking with those for the crystal structure of warfarin–HSA (PDB: RWF/PDB ID: 2BXD) and phenylbutazone–HSA (PhB/PDB ID: 2BXP) it was found that the both RWF and PhB had some similar interactions with those of these ligands. It can be noticed in case of 6-MP, that when 6-MP was individually docked to detected cavity, the hydrogen bonds formed with Arg257 residue were important for binding of both neutral (Fig. 5a) and anionic (Fig. 5b) forms. However, the orientation of the both poses of 6-MP inside the binding site was different. The preferred pose conformation of neutral 6-MP was positioned to form additionally the hydrogen bond with Tyr150, while the negatively charged N(1) atom of anionic form of 6-MP was bonded by a salt bridge to side chain of Lys199. The only N(3) and N(9) atoms of 6-MP molecules as acceptors were involved in hydrogen bond interactions.

**Fig. 6:**
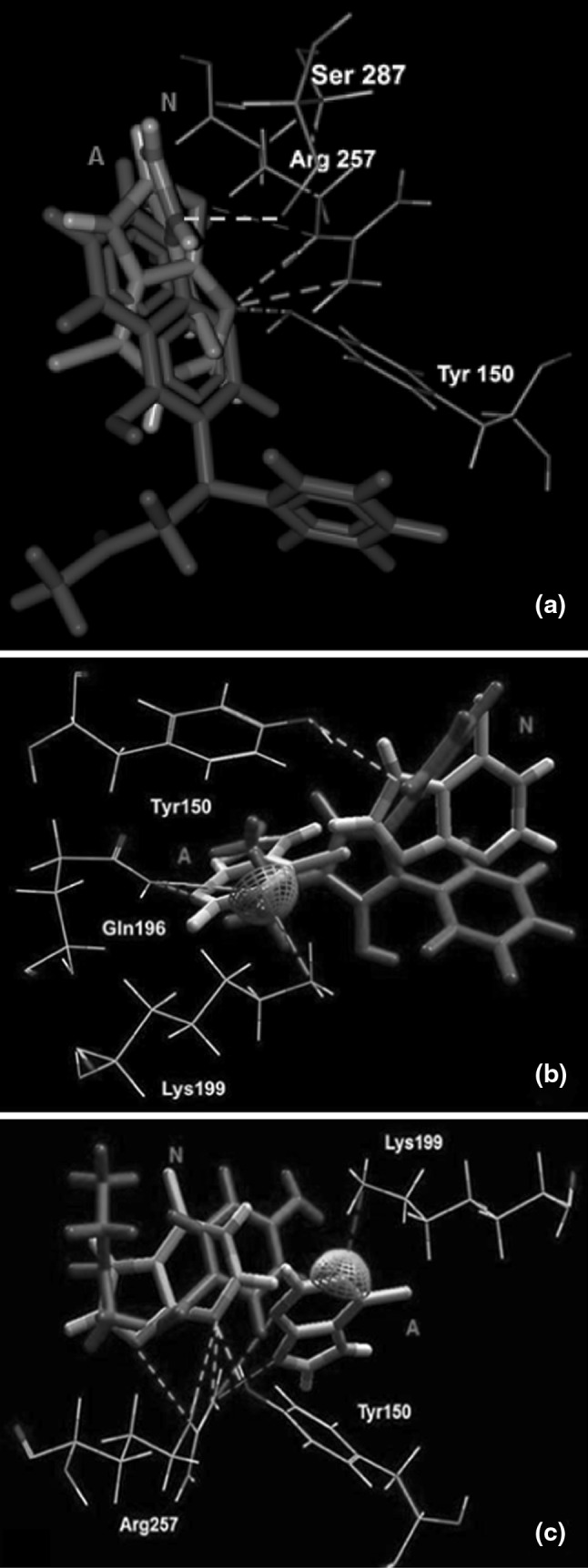
Molecular visualization of binding of 6-MP with RWF–HSA complex (**a**), PhB–HSA complex (**b**) and ABE–HSA complex (**c**). The marker–HSA complexes, which are also presented in Fig. 4 a–c were obtained by using MVD [37] program. Only the hydrogen bonds and electrostatic interactions between 6-MP and amino acids residues are presented. Hydrogen bonding interactions are shown in *dashed green lines*, strong electrostatic interactions are visualized as partial spheres oriented in the direction of the interaction and as *dashed green lines*. The ligands and amino acids are shown in a *stick* and *thin stick* representation, respectively and are *colored* according to element type (in the CPK *color* scheme). N and A, neutral and anionic form of 6-MP, respectively (Color figure online)

Based on the experimental work the possibility of displacement of 6-MP by RWF and PhB and simultaneous binding in presence of ABE has been raised. Therefore, the binding mode of 6-MP with HSA also was studied using the obtained structures of the RWF–HSA, PhB–HSA and ABE–HSA complexes. The results of docking of 6-MP to marker–HSA complexes are presented in Fig. 6. Comparing the interactions between 6-MP and HSA when 6-MP was docked to unoccupied cavity (Fig. 5) with those between 6-MP and HSA when 6-MP was docked into cavity when RWF (Fig. 6a) or PhB (Fig. 6b) were bound, it was found that 6-MP had other interactions if markers were present in binding site. The orientation of 6-MP in binding site depends upon RWF and PhB. Thus when the RWF or PhB were present in binding site, they seemed to impede the access of 6-MP to Lys199 or Arg257, and other residues were capable for interacting with 6-MP. The differences in presence of RWF were that neutral form of 6-MP, exited a specific hydrogen bond with Arg257 and new hydrogen bond with Gln196 was identified. Breaking the salt bridge between the anion of 6-MP and positively charged Lys199, resulted in formation of new hydrogen bond with Tyr150. Also in the presence of PhB the Arg257 residue was unable to binds with the both forms of 6-MP. The 6-MP as a neutral molecule had only one hydrogen bond interaction with Tyr150, while for anionic form of 6-MP the hydrogen bonds with Arg257 were replaced by hydrogen bond with Gln196. The molecular docking calculations summarized in Table 3 (part B and C) indicating that 6-MP had less favorable interactions with HSA than the 6-MP docked to unoccupied cavity (Table 3, Part A). If the 6-MP was docked to ABE–HSA complex, then the observed interactions for both forms of 6-MP were retained (Fig. 6c) and the all binding energies were the same (Table 3, part D). It therefore appears that the binding mode of 6-MP was independent of the presence of ABE.

**Fig. 7:**
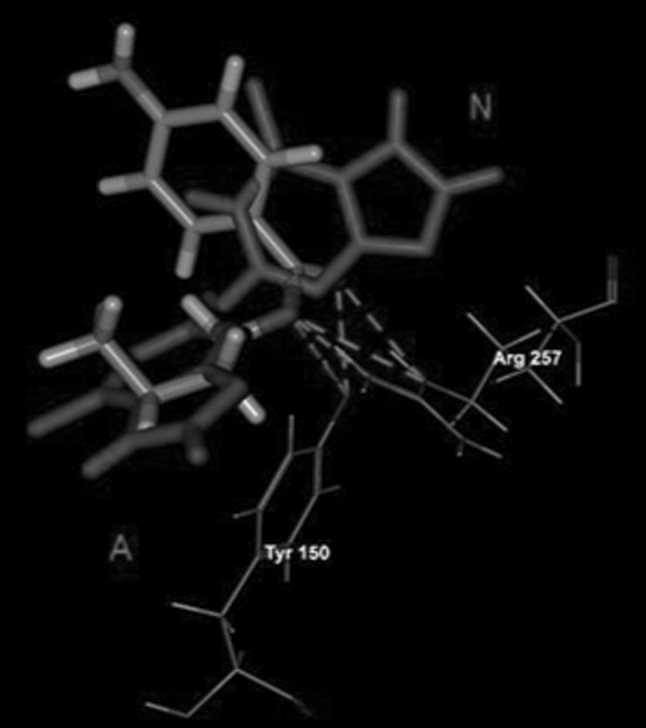
Molecular visualization of binding of 6-ABE with 6-MP–HSA complex obtained by using MVD [37] program. Only the hydrogen bonds and electrostatic interactions between ABE and amino acids residues are presented. Hydrogen bonding interactions are shown in *dashed green lines*. The ABE and amino acids are shown in a *stick* and *thin stick* representation, respectively and are *colored* according to element type (in the CPK *color* scheme). The 6-MP is* colored red*. N and A, neutral and anionic form of 6-MP, respectively (Color figure online)

The results from displacement experiment suggested that ABE was binding stronger to HSA in the presence of 6-MP. In order to explain this observation the ABE was also docked to 6-MP–HSA complex. It was seen that ABE was binding to 6-MP–HSA complex, further stabilized by five hydrogen bonds. Comparing the interaction between ABE and HSA it was noteworthy, based on the estimated binding energies (Table 3, part E), that in spite of small difference between the E-Total for ABE docked to empty cavity and docked to cavity when 6-MP was bound, the hydrogen bonds energy was found to be 2.5-fold larger than that for ABE docked individually. The new stronger hydrogen bonds were involved between keto and oxo groups in ABE and charged guanidinium group of Arg222 residue and O–H group of Tyr150 (Fig. 7).

## Discussion

Human serum albumin is a major protein component of blood plasma and due to its endogenous and exogenous binding properties plays an important role in the distribution, metabolism, elimination and therapeutic effectiveness of drugs. The principal binding regions of albumin are located in subdomain IIA and IIIA which are structurally characterized by the presence of the hydrophobic interior and the polar exterior. The binding cavity in subdomain IIIA possesses the primary binding activity whereas the cavity in subdomain IIA is more specialized. The albumin may accommodate a very wide range of compounds. HSA binds mainly weakly acidic (anionic) drugs, though it also binds with certain basic and some neutral drugs. Ligands binding to site I are often generally bulky heterocyclic compounds with a negative charge localized in the middle of the molecule [13, 18]. Kragh-Hansen et all. [31] showed, that the site I must be large, because the big molecules as for example bilirubin can be bound. The fact that very wide range of compounds with different chemical structures bind to the region with high affinity indicates that the site is adaptable, very flexible and rapidly changing in shape. Some of these changes are intrinsic, but most of them are related to the binding of ligands. However, different authors suggest that albumin has limited number of binding sites [15, 29]. Since, the number of protein binding sites is limited, the potential interaction between two drugs that simultaneously bind to albumin will exist. Binding of drugs to plasma protein controls their free (active) concentration and provides a reservoir for a longer action [2]. The pharmacological effect of the increase in unbound drug concentration will be increased elimination of drugs excreted by glomerulal filtration. If the displaced drug diffuses to more remote sites and less drug is transported to the sites of elimination in the liver and kidney, displacement may cause a short-lived increase in its half-life [34]. For example phenylbutazone can displace warfarin from albumin binding site. The clinical studies showed that the eliminations half-life of warfarin is decreases, while its anticoagulant activity is enhanced, by concurrent administration of phenylbutazone [22, 44]. In general, simple displacement interactions are clinically unimportant. However, the combination of plasma protein displacement and decreased free drug clearance will often result in a serious interaction [44]. For all of these reasons, it is important to have a good understanding of how drugs bind to serum albumin and of how these interactions are affected by other substance.

The aim of this study was to examine the binding of 6-MP to HSA. The interaction between 6-MP and HSA was investigated under physiological pH 7.4 in vitro using circular CD and equilibrium dialysis, and in silico by molecular docking. Far-UV CD is a suitable method for the study of secondary structure of protein. The interaction between albumin and ligands often is accompanied by detectable conformational changes of the protein [28]. Therefore the CD may be also employed for determination of the effect of ligand on the secondary structure of the protein upon binding with the ligand. The CD parameter at the wavelength 222 nm of the spectrum of HSA is proportional to the α-helix content. Hence, the decrease or increase in the negative value of this parameter corresponds to changes of a α-helix secondary structure of the protein and may provide a direct information on the binding interaction and the formation of the ligand–protein complex. Here, the influence of the 6-MP on the secondary structure of the HSA was studied by CD spectra in far-UV region. When 6-MP was added to the solution of native HSA, the negative band intensities at 222 nm decreased relative to the spectra in the absence of 6-MP. The helical content decreased from ~55 to ~53 % at low concentration of 6-MP and to ~50 % at higher concentration of 6-MP (Fig. 2; Table 1). The observations than can be made from this experiment were that in the presence of 6-MP the protein secondary structure was partially disordered with a loss of α-helical stability due to 6-MP–HSA complex formation.

Albumin is known to be able to bind numerous endogenous and exogenous compounds mostly through the formation of non-covalent complex at specific binding sites. The binding sites have configurational adaptability because the interhelical attractions in albumin are week, permitting the amino acids residues to assume different orientations. As binding occurs, the helices separate, and new binding sites are created [46]. However, two different models can be taken into consideration. The Scatchard [42] model assumes that albumin contains a fixed number of pre-existing sites that compete independently for available ligand, and the Karush [23, 24] model which assumes that binding sites are created and modified as binding occurs, accounting for conformational changes and cooperative binding effects [46]. The results obtained from the many studies performed on serum albumin by Kragh-Hansen et all. [30] also showed that concomitant ligand binding usually is only partially competitive and may include allosteric effects. However, when analysing ligand–ligand displacement interactions, the possibility of displacement of ligand from one site to another site should be taken into account [31, 40].

In order to determine the specificity of the 6-MP binding and location of the 6-MP binding site on HSA, the displacement experiment was carried out using RWF, PhB and ABE which are known to bind at region Ia, Ib and Ic of site I, respectively. The binding of 6-MP to HSA in the absence and presence of these site markers was investigated by analyzing the changes of the HSA-bound fraction of 6-MP and site markers (Table 2). It was evident from the simultaneous binding data that the 6-MP could bind in site I, because it was displaced which was indicated by decrease of its bound fraction from ~23 to ~18 %, in the system 6-MP–RWF and 6-MP–PhB (Table 2). However, the 6-MP did not decrease the ability of the RWF and PhB to bind. This may be explained in two ways. The 6-MP displacement (p%) by the RWF and PhB was not increased up the level expected for a competitive mechanism. Hence, the decreased binding of 6-MP after the addition of RWF or PhB, but not vice versa, indicated displacement of 6-MP rather by non-cooperative mechanism, than the direct competition between 6-MP and RWF or PhB. It cannot be excluded, that the portion of 6-MP displaced from the site I by RWF or PhB, rebound to its another binding site. Then it would be possible that the displacement of 6-MP by RWF or PhB was caused in a competitive manner and then the observed displacement which was less than that expected for direct competitive model would have been explained.

The observed little effect of ABE on the binding of 6-MP indicates that the both ABE and 6-MP did not compete for a common binding site and could bind to separate and independent binding sites. Moreover the enhancement of ABE binding in presence of 6-MP could be explained with the results obtained from CD measurement. As it was stated on the basis of CD spectra (Fig. 2; Table 1), the secondary structure of HSA was changed upon the interaction with 6-MP. If these changes were around the binding site of ABE in region Ic, they could change its shape thereby increase the affinity of HSA for ABE at the site in this region. If, 6-MP–induced changes in the binding site caused a marked increase in ABE binding to site, it may be concluded that the improvement of the binding of ABE by 6-MP was via a positively cooperative mechanism.

The docking simulation by using the MVD program was appropriate for clarifying the binding mode of 6-MP. It was found that the four polar residues of Tyr150, Lys199, Arg222 and Arg257, within 6 Å distance of the docked ligands influenced binding of 6-MP and site markers (Figs. 4, 5). Independently from other interactions, these with the residues of Arg257 were required for binding of all the ligands. The individual docking of the ligands showed that they all could bind to HSA through the electrostatic interaction (exception ABE) and hydrogen bonding interaction. However, most of the binding energy which stabilized the interactions comes from steric interactions between ligands molecules and unchanged amino acids residues which were present in proximity (around 6 Å) of the docked compounds (Fig. 3; Table 3). The calculated E-total binding energy in order PhB > RWF > ABE > 6-MP (as an anion) >6-MP (as neutral) corresponded to the order of logK of these ligands obtained by equilibrium dialysis (Table 2). The 6-MP, likewise as RWF, PhB and ABE, was located within binding cavity of subdomain IIA and the space occupied by site markers overlapped with that of 6-MP. To assess the impact of site markers on 6-MP binding to site I, the 6-MP was docked to the marker-HSA complexes obtained by molecular docking. Due to the fact that the side chains of amino acids close to the detected cavity were allowed to changing during the docking simulation, in each case the two ligands could bind simultaneously into cavity. This means that both ligand-binding area and residues required for interaction at the binding site in were present to accommodate 6-MP in addition to accommodation RWF, PhB or ABE. This result and similarly effect observed in equilibrium dialysis experiment showed that the binding site had space and appropriate shape to accommodate two ligands. However, when the 6-MP was docked to the RWF–HSA or PhB–HSA complexes, then upon presence the both RWF or PhB, exhibited the other pattern of binding with site I (Fig. 6). The differences were that neutral form of 6-MP in presence of RWF and both form of 6-MP in presence of PhB lost a specific interaction with Arg257. The orientation and location of the 6-MP molecules were then probably different, because of another residues (Gln196 and Ser287) capable of interacting with 6-MP. The results suggested also that the microenvironment around 6-MP became more hydrophilic. The explanation was that 6-MP lost the hydrogen bonding interaction with Arg257, which is buried in hydrophobic core of HSA. As it was showed by equilibrium dialysis the binding of 6-MP was decreased, probably because of the higher affinity of RWF and PhB to HSA. The association constants for RWF (log K_exp_^HSA^ = 5.74) and PhB (log K_exp_^HSA^ = 5.70) were about 18 times larger than that for 6-MP (log K_exp_^HSA^ = 4.48) and PhB and RWF with higher affinity could displace the 6-MP causing the reduction of 6-MP affinity to HSA and increase it free concentration (Table 2). In contrast to RWF and PhB, the presence of ABE in binding site had not effect on the binding mode of 6-MP and the same residues required for binding of 6-MP existed in HSA even when it was bound to ABE-HSA complex. However, it was interesting to note, that when ABE was docked to 6-MP–HSA complex, then ABE received the new hydrogen bonds with Arg257 and the E-Total interaction energy with HSA was higher (Fig. 7; Table 3, part E). This can be explained by the fact that little conformational perturbations in the secondary structure of HSA demonstrated by CD spectra (Fig. 2, Table 1) could change the shape of binding site of ABE in region Ic thereby enhance the affinity of ABE to HSA.

Identical binding site with a different binding site between two ligands indicate a competition for the binding site between the two ligands in favor of ligands with higher affinity [22]. Hence, competition for the binding site between 6-MP and RWF or PhB was putative to occur in favor of the former two. However, the 6-MP displacement by the RWF or PhB was not up the level expected for a competitive mechanism, therefore displacement of 6-MP was rather by non-cooperative than that the direct competition. By contrast the tighter binding of ABE in presence of 6-MP could be due to the positively cooperative mechanism.

In summary, the results presented seem to show that 6-MP can interact with HSA in subdomain IIA and that 6-MP and site markers through wide and flexible ligand-binding site in subdomain IIA, could bind there with significant overlap. The RWF and PhB, but not ABE, may affect the binding of 6-MP to site I. However, HSA binding of ABE could be by a slight degree enhanced by the presence of 6-MP. The molecular docking simulation corroborate this finding. Although, the proteins in solutions are not static structures, and molecules change between different conformation, the obtained results from the in vitro experiments were in consonance with the results from in silico study and complement with each other. From a drug–drug displacement interactions point of view the results may be important when 6-MP is administered concomitantly with drugs containing warfarin belonging to the class of oral anticoagulants and phenylbutazone belonging to the class of non-steroidal anti-inflammatory drugs.

## Abbreviations

ABE: n-Butyl *p*-aminobenzoate
CD: Circular dichroism
HSA: Human serum albumin
6-MP: 6-Mercaptopurine
MVD: Molegro Virtual Docker
PDB: World wide web Protein Data Bank
PhB: Phenylbutazone
RWF: Warfarin
